# Rectus Abdominis Sheath Inversion Flap Reinforced With Polypropylene Mesh (RASIFRPM): A Reconstructive Alternative for the Management of Complex Ventral Hernia

**DOI:** 10.7759/cureus.114005

**Published:** 2026-08-05

**Authors:** Irma L Maldonado Barrios, Francisco J Barahona Browne, Luis A Cervantes Ruiz, Jorge A Ramirez Aguilar, Rafal L Smolinski Kurek

**Affiliations:** 1 Department of General Surgery, Hospital Regional de Alta Especialidad del Bajio, Leon, MEX

**Keywords:** abdominal wall flap, rectus abdominis, surgical flaps, surgical mesh, ventral hernia

## Abstract

Complex hernias require a staged approach to abdominal closure. In patients where primary fascial closure is not possible, several techniques have been proposed for early abdominal wall reconstruction.

Rectus abdominis sheath inversion flap reinforced with polypropylene mesh (RASIFRPM) is a reconstructive technique for complex abdominal hernias that combines bilateral release of the rectus abdominis sheath with its medial mobilization and prosthetic reinforcement. We present a case of a hernia with loss of domain that required early reconstruction to achieve complete abdominal wall closure.

## Introduction

Complex ventral hernia repair remains one of the greatest challenges in general surgery, as both recurrence and postoperative complication rates are high in patients with large fascial defects or a history of multiple previous abdominal operations [1,2].

The primary objective of abdominal wall reconstruction is to achieve a durable, tension-free fascial closure while restoring the native midline anatomy through medialization of the rectus abdominis muscles [2,3].

Within this context, techniques such as the rectus abdominis sheath inversion flap reinforced with polypropylene mesh (RASIFRPM), known in Spanish as Colgajo de Inversión de la Vaina Anterior del Recto Reforzado con Malla de Polipropileno (CIVARRM), are based on the principle of using autologous fascial rotation flaps to bridge the midline defect, thereby providing a functional reconstruction when conventional primary fascial closure cannot be achieved [4].

The RASIFRPM/CIVARRM technique is a reconstructive approach for complex ventral hernias that combines bilateral release of the anterior rectus sheath with medial advancement and prosthetic reinforcement. Primary fascial closure is achieved by inverting the anterior rectus sheaths toward the midline, creating a continuous fascial-muscular layer that restores abdominal wall integrity.

This reconstructive strategy is further strengthened by polypropylene mesh reinforcement, which provides additional structural support by distributing tensile forces across the abdominal wall. This approach is consistent with current international recommendations advocating mesh reinforcement in the repair of large ventral hernias, regardless of whether primary fascial approximation is feasible [3]. Furthermore, the literature emphasizes that both the use of prosthetic material and its appropriate positioning and secure fixation are critical factors in minimizing hernia recurrence [5].

This technique is particularly valuable in patients with large abdominal wall defects, loss of domain, or recurrent ventral hernias in whom standard fascial closure is not feasible.

Within the context of abdominal wall reconstruction and open abdomen management, the loss of domain refers to the condition in which, following prolonged open abdomen management or the development of a massive ventral hernia, progressive lateral retraction of the fascial edges occurs. As a result, a substantial proportion of the abdominal viscera no longer resides within the native abdominal cavity, creating an increasingly larger defect that complicates definitive fascial closure [5].

Anterior rectus sheath inversion flap is a surgical technique originally described to facilitate early fascial closure in patients requiring open abdomen management. The procedure consists of mobilizing and inverting the anterior rectus sheath to bridge the fascial defect, thereby reducing the need for split-thickness skin grafting and facilitating subsequent definitive abdominal wall reconstruction [4].

Preperitoneal mesh placement is most commonly described as sublay (retrorectus) mesh placement. In this technique, the prosthetic mesh is positioned posterior to the rectus abdominis muscle and anterior to either the posterior rectus sheath or the peritoneum. This location is preferred because it prevents direct contact between the mesh and the abdominal viscera, thereby reducing the risk of adhesions and enterocutaneous fistula formation. It is considered a reliable method for reinforcing the midline and evenly distributing tensile forces during complex abdominal wall reconstruction [5].

## Case presentation

A 36-year-old man with a history of multiple abdominal operations presented for evaluation of a recurrent post-incisional ventral hernia. His relevant surgical history included a laparoscopic biliary-enteric diversion with hepaticojejunostomy performed in November 2021 for a Todani type I choledochal cyst. His postoperative course was complicated, requiring emergency exploratory laparotomy for massive hemoperitoneum, repair of a biliary-enteric anastomotic leak, abdominal washout, drain placement, intensive care unit admission, and additional abdominal wall procedures. During follow-up, the patient developed a post-incisional ventral hernia, which was initially repaired by an open primary fascial closure at another institution. However, subsequent follow-up demonstrated hernia recurrence with progressive enlargement of the abdominal wall defect.

**Figure 1:**
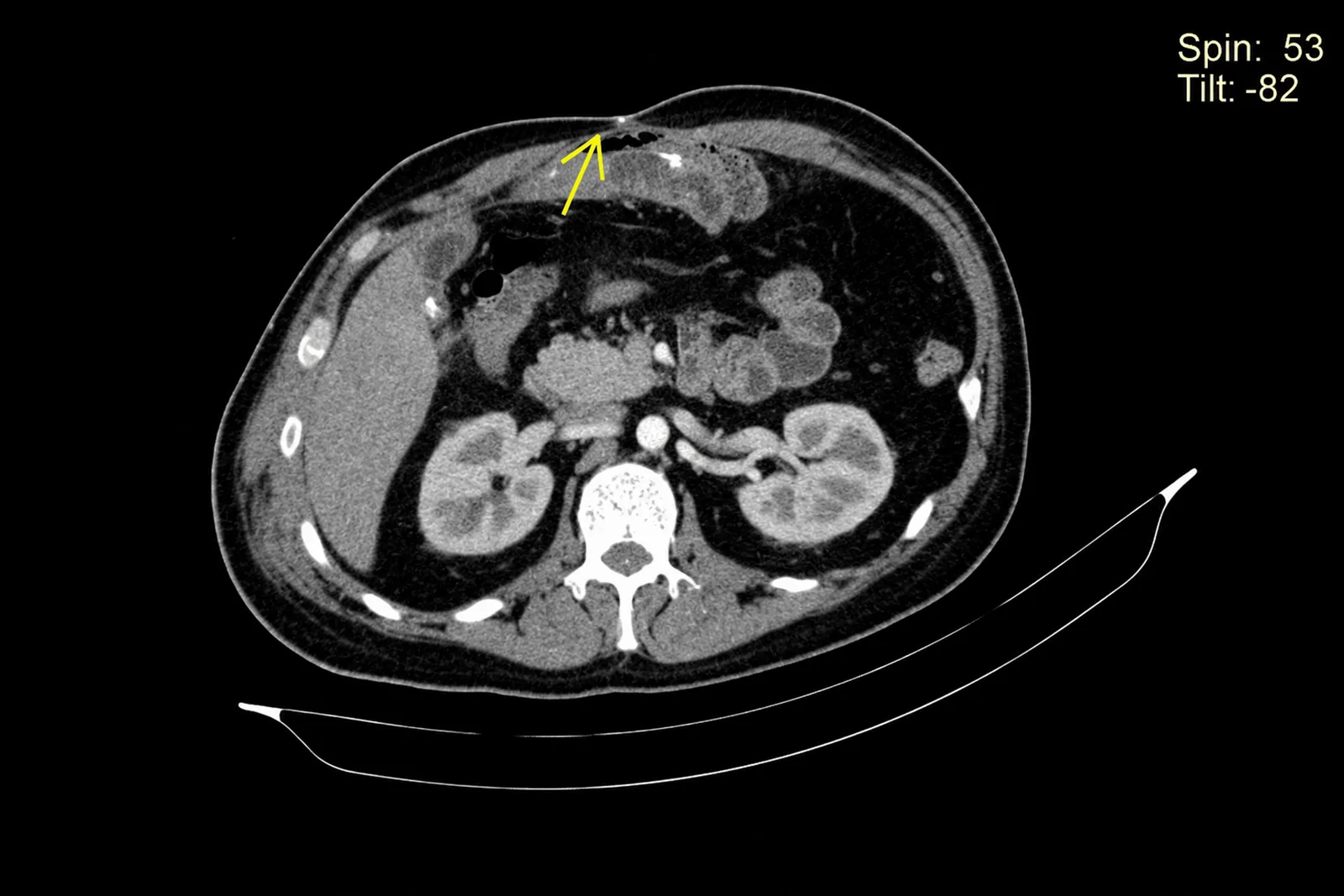
Preoperative CT of the abdominal wall defect. Axial CT image at the level of the left renal hilum shows the upper component of the abdominal wall defect. The arrow shows the precise location of the hernia. CT: computed tomography

**Figure 2:**
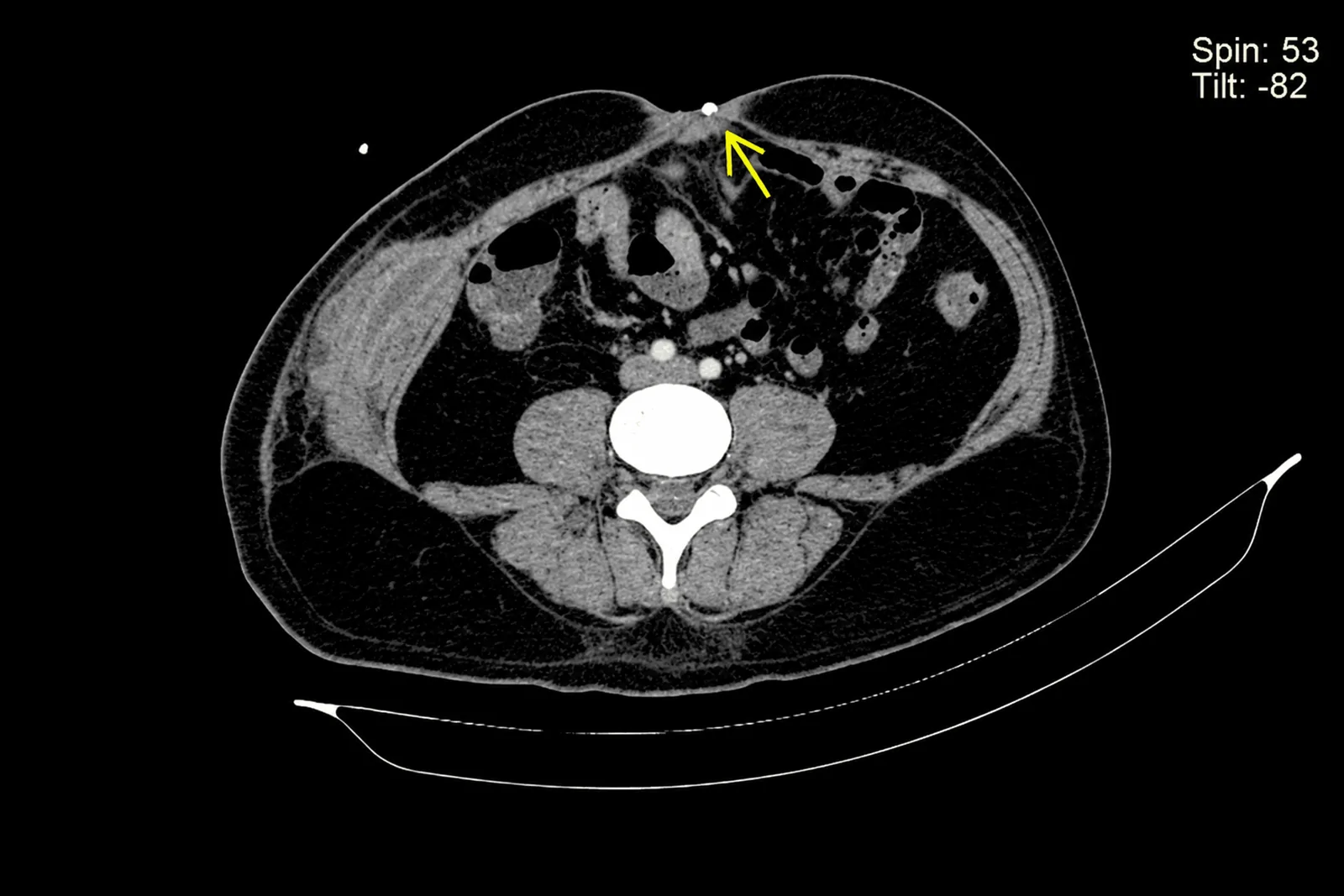
Caudal axial computed tomography image additionally showing the defect at a lower level. The arrow shows the precise location of the hernia.

**Figure 3:**
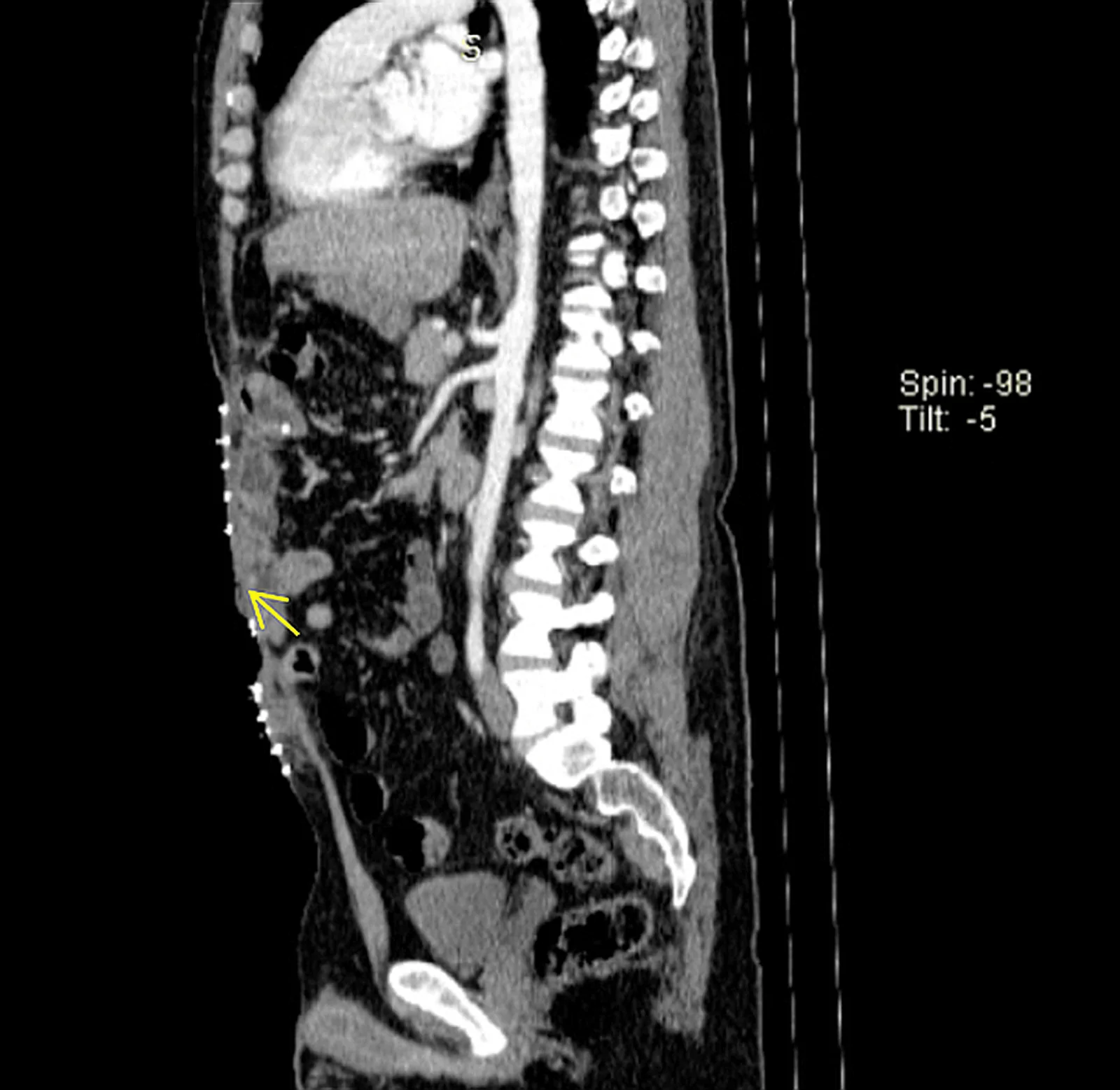
Sagittal computed tomography image showing the craniocaudal view of the hernia without radiological evidence of loss of domain. The arrow indicates the precise location of the hernia

The patient was admitted on February 16, 2023, with a diagnosis of recurrent post-incisional ventral hernia, classified as M1-M4, W3. Due to the large abdominal wall defect, history of multiple previous abdominal procedures, and recurrent nature of the hernia, an elective abdominal wall reconstruction under general anesthesia was planned. The preoperative computed tomography (CT) scan showed a large midline ventral abdominal wall defect with no radiological evidence of loss of domain; therefore, no preoperative conditioning measures were performed (Figures 1-3).

**Figure 4:**
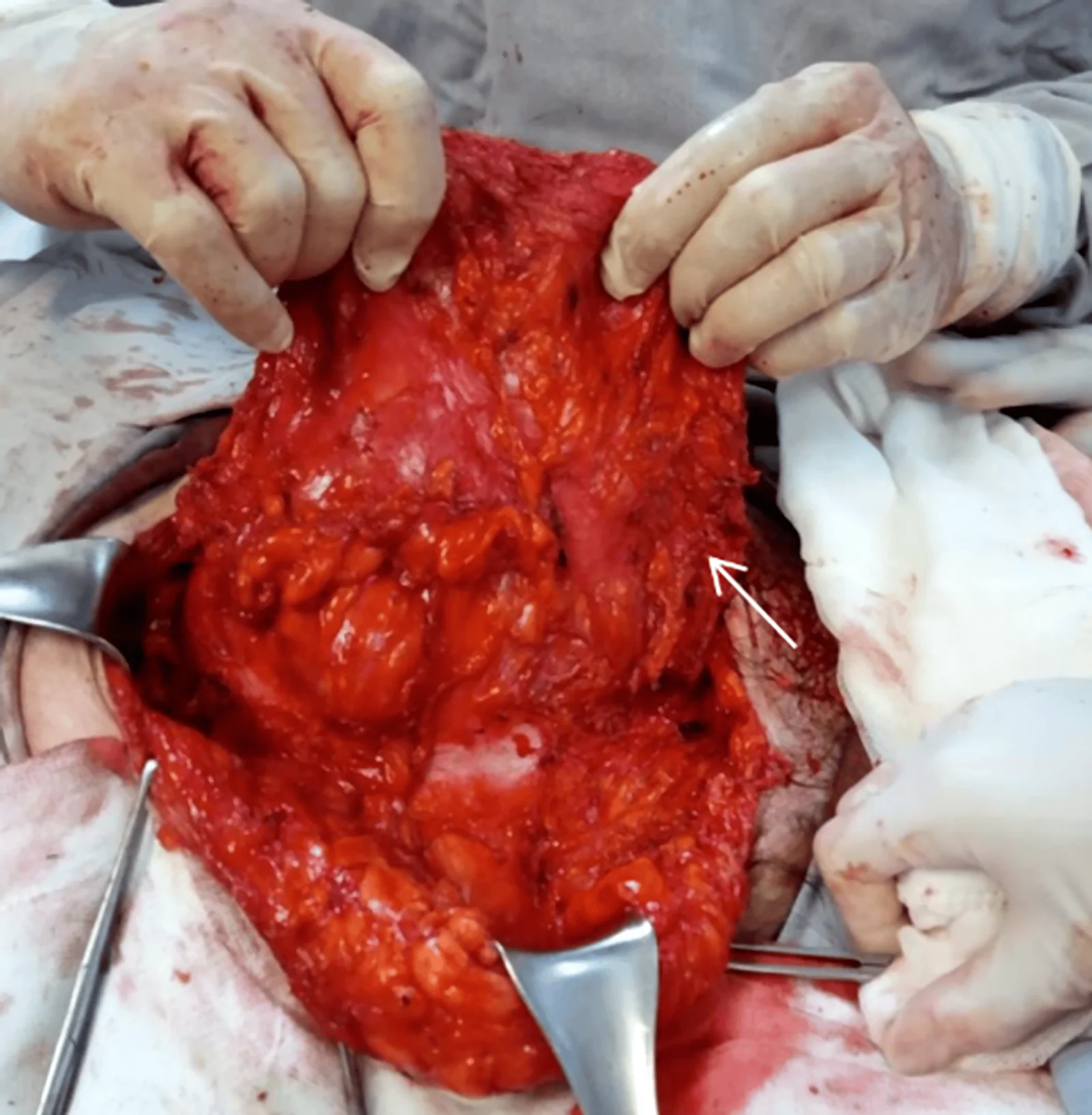
Intraoperative sequence of the RASIFRPM technique. The edges of the hernial defect were identified, and the hernial sac was exposed (indicated by the arrow). RASIFRPM: rectus abdominis sheath inversion flap reinforced with polypropylene mesh

On February 17, 2023, an abdominal wall reconstruction was performed using an anterior rectus sheath turnover (inversion) flap reinforced with a preperitoneal polypropylene mesh. An elliptical incision was made over the previous midline scar. Dissection was extended through the abdominal wall planes until the hernial sac was identified (Figure 4).

**Figure 5:**
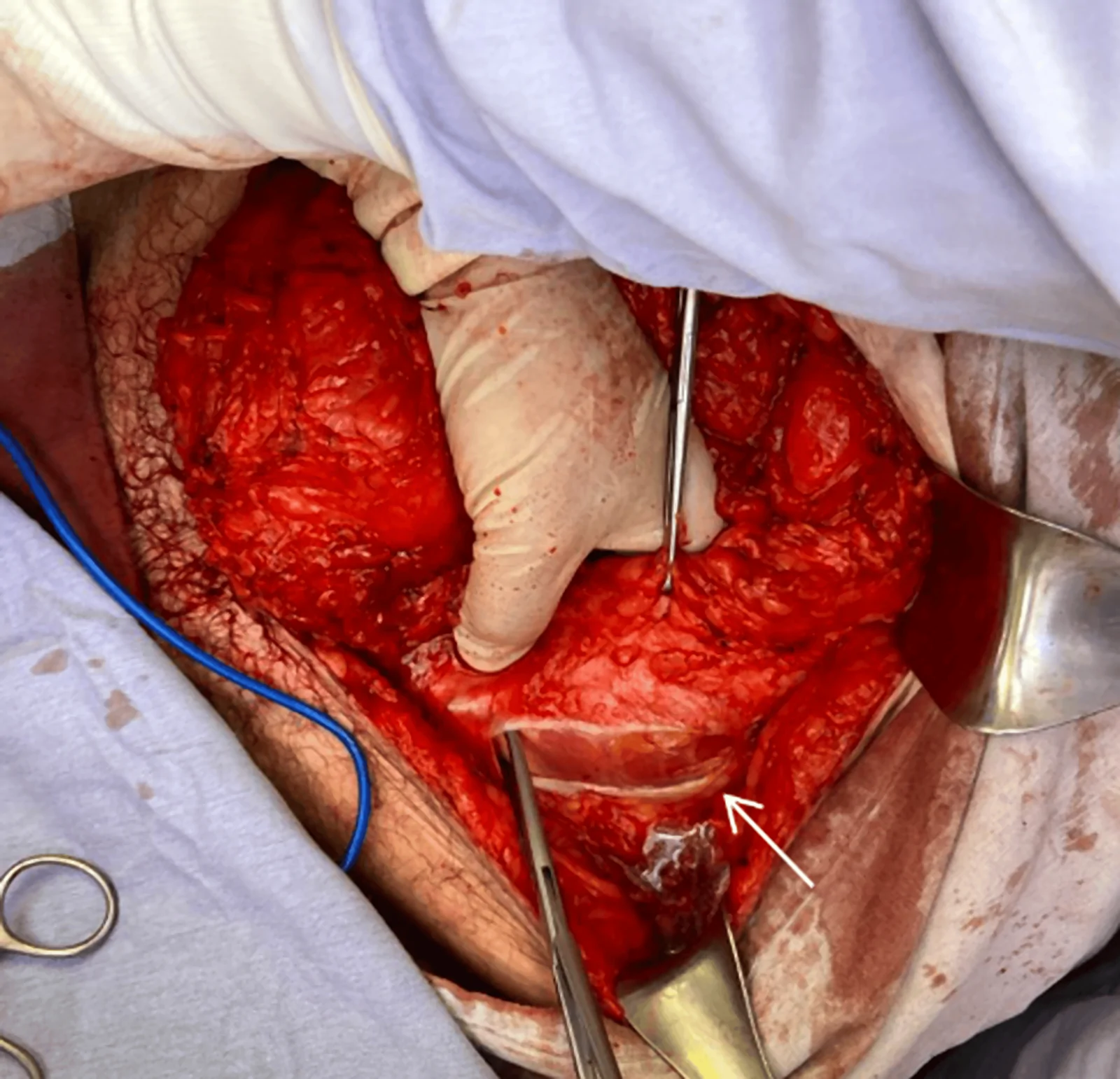
Incision of the anterior rectus sheath near its lateral border (arrow) to initiate the creation of the turnover flap (anterior rectus sheath flap after lateral to medial dissection).

The sac was dissected, and the medial and lateral borders of the rectus abdominis muscles were identified. A preperitoneal plane was developed over the aponeurotic plane. Next, the anterior rectus abdominis sheath was incised bilaterally at its lateral border, and the anterior sheath was mobilized medially to create a turnover flap (Figure 5).

**Figure 6:**
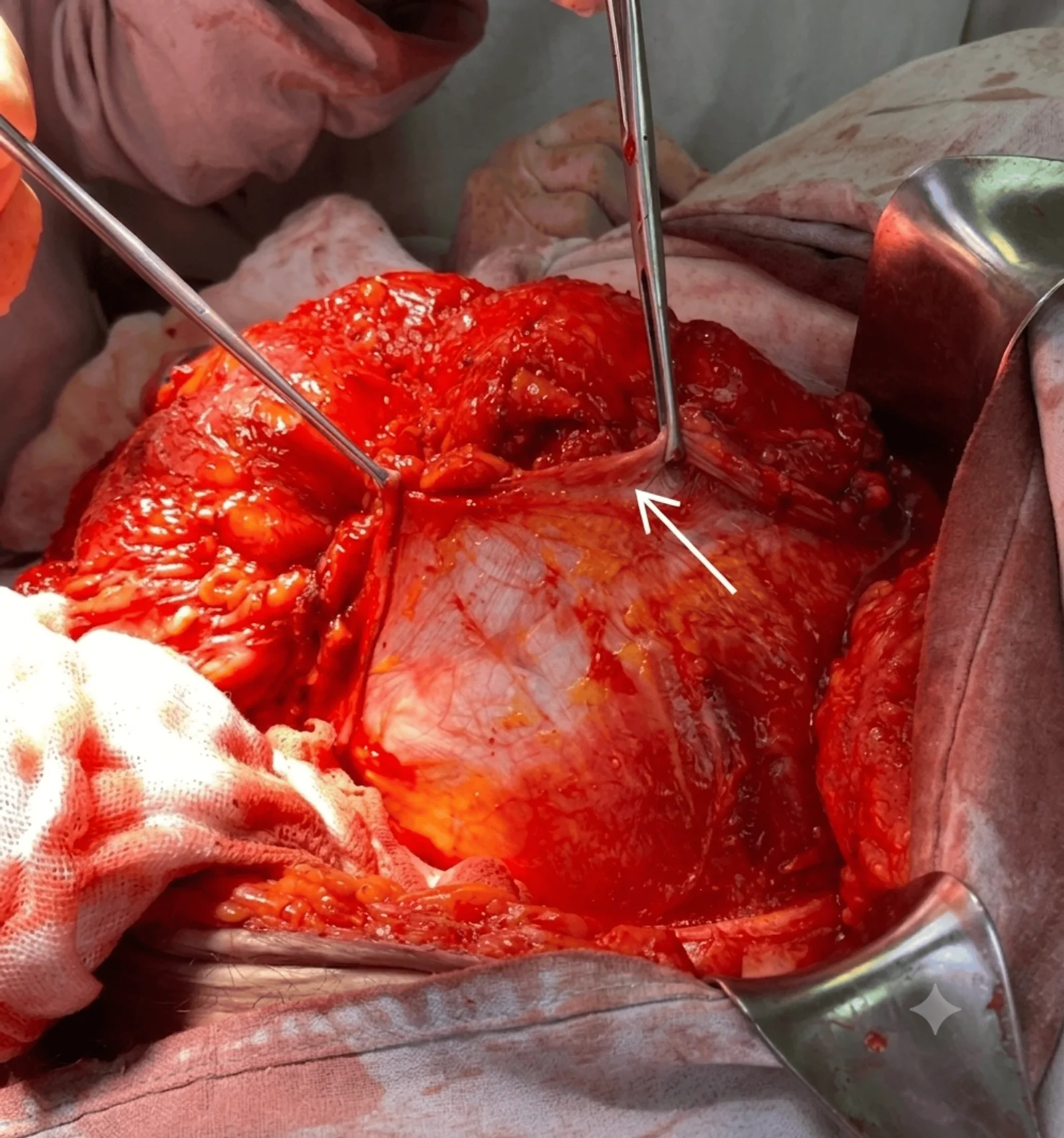
Fully mobilized anterior rectus sheath flap (arrow) after lateral to medial dissection.

The sheath was mobilized from lateral to medial, exposing the rectus abdominis muscle and the inner surface of the anterior rectus sheath flap (Figure 6).

**Figure 7:**
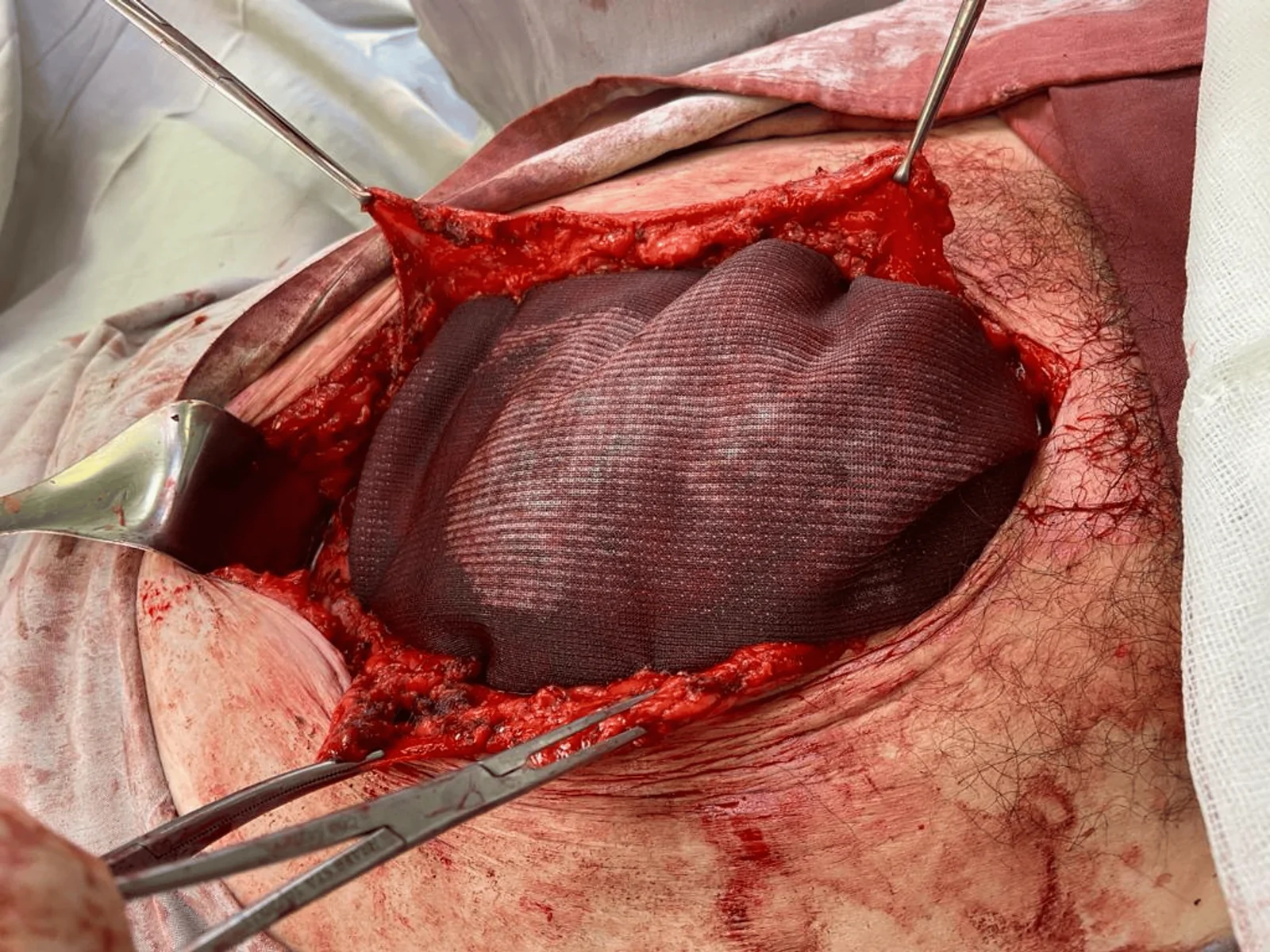
Placement of a 30 × 30 cm polypropylene mesh beneath the anterior rectus sheath flaps.

Before closing the fascial flap, a 30 x 30 cm polypropylene mesh was placed in the preperitoneal position and secured with 0 polydioxanone U-sutures (Figure 7).

**Figure 8:**
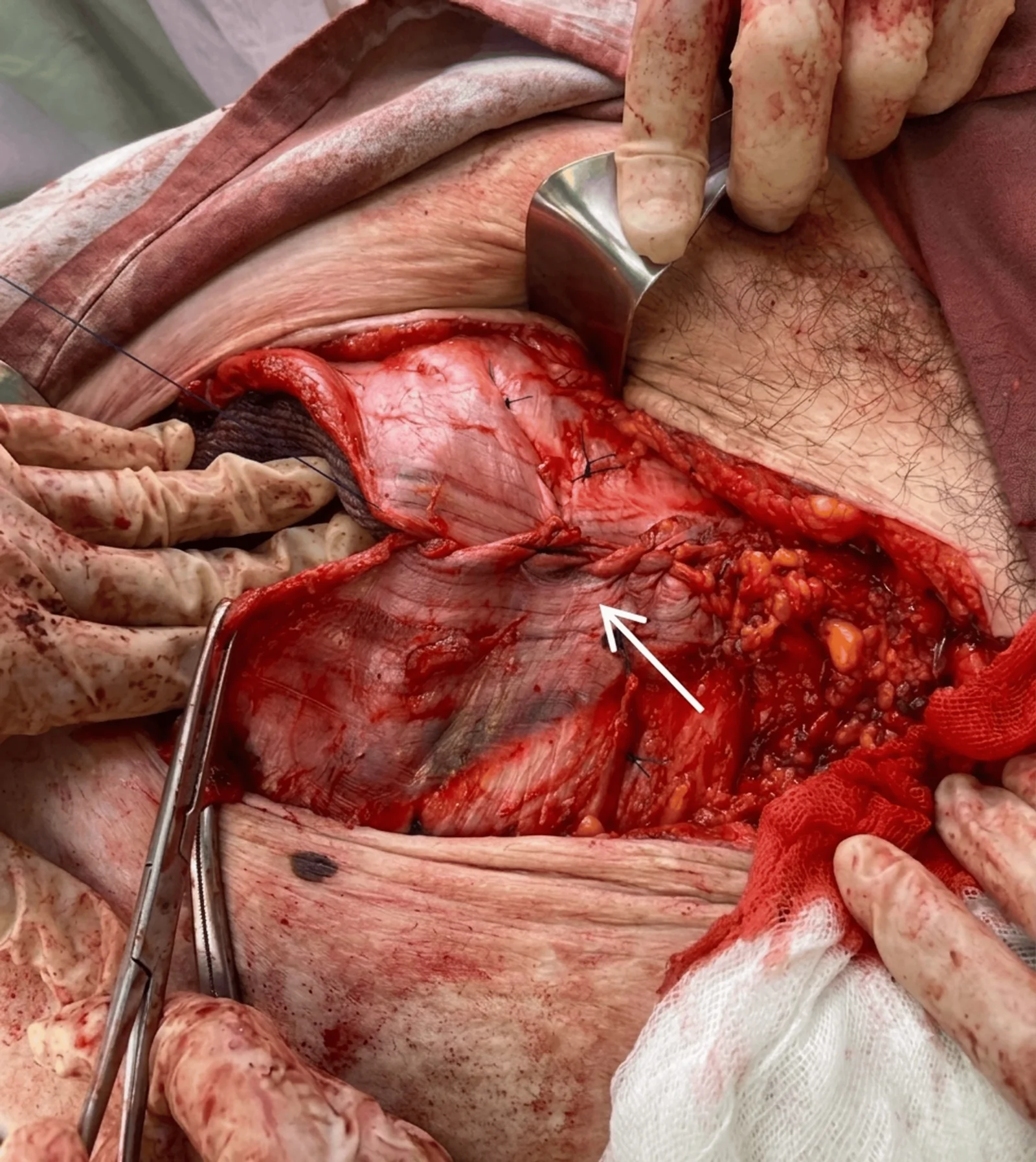
Final approximation of the inverted anterior rectus sheath flaps (arrow) after closure with a continuous suture.

The inverted anterior rectus sheath flaps were approximated with a continuous 0 polydioxanone suture, achieving midline reconstruction and complete aponeurotic coverage of the mesh (Figure 8). Two 19 Fr Blake drains were exteriorized through bilateral counter-incisions and connected to closed suction reservoirs. The subcutaneous tissue was closed with 2-0 polyglactin suture, and the skin was closed with surgical staples.

Intraoperative findings included a large ventral hernia, 25 cm in length and 18 cm in width, with normal-quality aponeurosis. No intraoperative incidents were reported. Estimated blood loss was 600 mL, and the surgical count was complete.

The immediate postoperative period was uneventful. The patient remained hemodynamically stable, with adequate pain control and tolerance to oral intake. Before discharge, the abdomen was soft and depressible, with no abdominal guarding or signs of peritoneal irritation. Both Blake drains showed minimal serosanguineous ​​​​​​output. The patient was discharged on February 18, 2023, with instructions for wound care, drain care, and avoidance of heavy lifting. Early postoperative follow-up showed an adequate postoperative course, with no evidence of wound dehiscence or hernia recurrence.

## Discussion

The reconstruction of complex abdominal wall defects remains one of the greatest challenges in reconstructive surgery because it requires the simultaneous restoration of fascial integrity, preservation of the dynamic function of the abdominal musculature, and adequate soft tissue coverage [1].

The primary goal of any abdominal wall repair is to achieve a durable, functional, and tension-free closure, while minimizing the risk of postoperative complications and hernia recurrence. In this context, RASIFRPM/CIVARRM has emerged as a reconstructive alternative that combines the principles of autologous tissue reconstruction with the mechanical reinforcement provided by a synthetic mesh [1,6].

Current evidence demonstrates that mesh-reinforced primary fascial closure is associated with lower recurrence rates than suture-only repairs. Furthermore, the concept of functional abdominal wall reconstruction emphasizes that restoring the midline improves force transmission between both hemiabdomens and facilitates the recovery of normal abdominal wall biomechanics [2].

The RASIFRPM procedure, as described in the present case of a patient with a recurrent ventral hernia, includes several critical technical steps, among which the precise identification of the medial borders of the rectus abdominis muscles and the development of the preperitoneal plane are essential. The fundamental principle of the RASIFRPM technique is the creation of a rotation flap, in which the anterior rectus sheath is incised longitudinally near its lateral border. Next, the anterior fascial layer is mobilized from lateral to medial, exposing the rectus muscle and allowing the fascia to be reflected toward the midline while using the medial border of the rectus muscle as structural support [6]. This advancement of autologous tissue allows for the closure of large abdominal wall defects, as demonstrated in the present case, without generating excessive tension that could compromise tissue perfusion [2,6].

To ensure the long-term durability of the repair, the rotation flap is reinforced with a polypropylene mesh. In this case, a 30 × 30 cm polypropylene mesh was placed in the preperitoneal plane and secured with non-absorbable sutures. Finally, the inverted fascial flaps were approximated at the midline using a continuous suture, achieving complete, tension-free, and mechanically robust aponeurotic coverage.

One of the main advantages of the RASIFRPM technique is that it allows for the medialization of the rectus muscles and the reconstruction of the linea alba with minimal tension, thereby improving the dynamic function of the abdominal wall and reducing recurrence rates, which have been reported between 5.1% and 7.3% [1].

Synthetic mesh remains the current gold standard for ventral hernia repair due to its high tensile strength, excellent tissue integration, and cost-effectiveness. Several systematic reviews have shown that placing the mesh in the retromuscular plane significantly decreases the incidence of infectious complications, while improving the long-term durability of the repair [7]. The restoration of fascial continuity allows for an adequate redistribution of the forces generated by intra-abdominal pressure, thus reducing tension along the suture line. Repairs utilizing the mesh as a bridging technique without primary fascial closure have consistently shown higher recurrence rates than those combining fascial closure with mesh reinforcement [6]. These findings support the rationale for mesh reinforcement in the RASIFRPM technique, whose contemporary reconstructive principle is to achieve primary fascial approximation whenever feasible and use the prosthetic mesh solely as a reinforcement rather than a bridge [8].

A 2026 review article evaluating the use of autologous flaps in the repair of complex hernia defects documented a subgroup study utilizing rectus abdominis sheath flaps, which comprised 17 studies and 180 patients, with promising results. Flap viability was 100%, recurrence was 3.3%, and the return to daily activities was 73.8% at six months post-procedure; furthermore, the surgical technique demonstrated versatility in cases of emergency laparotomy, surgical site infections, and malignancy [9,10].

Overall, the available evidence supports the surgical principles of the RASIFRPM technique. However, prospective studies with larger patient cohorts and longer follow-up are needed to more precisely define its recurrence rates, postoperative complications, and functional outcomes compared to other established abdominal wall reconstruction techniques.

## Conclusions

The RASIFRPM technique represents a safe, reproducible, and accessible alternative for the management of complex ventral hernias. By using the patient's own anterior abdominal sheath as an autologous biological reinforcement, a firm and functional midline closure is achieved. As was observed in the case report, the combination of adequate aponeurotic mobilization and the mechanical support of the polypropylene mesh could be a promising surgical option for abdominal wall reconstruction in complex ventral hernias in selected patients. However, future studies with a larger number of patients and long-term follow-up are needed to confirm the safety and efficacy of the technique.
